# Lanthanum Chloride Causes Neurotoxicity in Rats by Upregulating miR-124 Expression and Targeting PIK3CA to Regulate the PI3K/Akt Signaling Pathway

**DOI:** 10.1155/2020/5205142

**Published:** 2020-05-05

**Authors:** Linlin Zheng, Jinhui Zhang, Shengjin Yu, Zhe Ding, Heling Song, Yan Wang, Yicui Li

**Affiliations:** ^1^Institute of Molecular Medicine, Medical School, Eastern Liaoning University, Dandong Liaoning 118003, China; ^2^Department of Nursing, Medical School, Eastern Liaoning University, Dandong Liaoning 118003, China; ^3^Department of Basic Medicine, Medical School, Eastern Liaoning University, Dandong Liaoning 118003, China

## Abstract

**Background:**

Lanthanum (La) exposure can cause central nervous system (CNS) damage and dysfunction in children, seriously affecting intellectual development. miR-124 plays an important role in the development of the nervous system. We exposed rats to a La environment then observed the rats' learning and memory damage and neurotoxicity and the relationship with miR-124.

**Methods:**

Rats were exposed to LaCl_3_ via drinking water. The rats' offspring were exposed to LaCl_3_ from their mother before weaning, then from La water for 28 days. A Morris water maze was used to observe spatial memory capabilities. H&E staining and TUNEL assays were used to observe pathological changes and apoptosis in the hippocampus. miR-124 was detected by RT-qPCR, and its targeting was confirmed by luciferase assay. The HT22 cell line was cultured with LaCl_3_ and treated with miR-124 mimics or inhibitors; then, expression of PI3K/Akt-related proteins was detected by western blot.

**Results:**

La exposure can lead to impaired learning and memory ability in offspring. Offspring with La accumulations in the hippocampus showed severe damage, disordered cells, and increased neurocyte apoptosis. In vitro, the postsynaptic density protein 95 was downregulated under La exposure and apoptosis increased. This effect of La can be attenuated by miR-124 inhibitors and enhanced by miR-124 mimics. LaCl_3_ exposure increased miR-124 expression and targeting on PIK3CA, downregulating PI3K, p-Akt, and p-NF-*κ*B p65.

**Conclusion:**

La causes neurotoxicity by upregulating miR-124 expression and targeting PIK3CA through the PI3K/Akt signaling pathway.

## 1. Introduction

Rare earth elements (REEs) consist of 15 elements including lanthanum (La), scandium (Sc), and yttrium (Y), usually found in symbiotic forms in the external environment. China, Russia, the United States, and Australia are the most REE-rich countries, with China ranking first. REEs have unique physical and chemical properties making them useful in agriculture, industry, pharmacy, and aquaculture. However, the accumulation of environmental REE residues seriously affects ecology and human health, and previous studies have confirmed that REEs can pass the blood-brain barrier and the fetal placental barrier. Young animals can also be exposed to REEs through breastfeeding, leading to damage and dysfunction of the central nervous system. As such, REE exposure has become a common health issue [1, 2].

La makes up a large proportion of the REEs found in nature. After La ingestion, increased La content was found in the serum and brain, suggesting La can pass through the blood-brain barrier and accumulate in the brain [3]. Increased La in the brain can impair development of the central nervous system and decrease neurological or behavioral function [4–6]. Chicks injected with high doses of La daily had significantly reduced learning ability [7], and rats exposed to La also showed learning and memory dysfunction in the Morris water maze [8, 9], indicating that La affects the central nervous system, especially damaging cognitive ability. Studies have shown that La may decrease learning ability and memory by affecting the distribution of trace elements in the brain, disrupting the dynamic balance of enzymes and neurotransmitter systems [10, 11]. These studies partially explain La neurotoxicity, but the mechanisms involved require additional in-depth study. Some researchers have found that La can lead to oxidative damage in the brain, causing morphologically and functionally abnormal hippocampal neurons and synapses. LaCl_3_ at 0.5-4.0 mmol/L can change the Ca^2+^ concentration in cells to induce apoptosis, leading to decreased learning and memory. Therefore, researchers believe that neuronal cell apoptosis and necrosis following La exposure are the pathophysiological basis of its neurological abnormalities.

The phospholipid inositol 3-kinase (PI3K)/protein kinase B (Akt) signaling pathway is one of the most important antiapoptosis pathways. A variety of neurotrophic factors inhibit apoptosis by activating the PI3K/Akt signaling pathway to play protective roles in the brain. The upstream gene phosphoinositide 3-kinase alpha (PI3KA) regulates the function of the PI3K/Akt pathway, leading to activation of RAS-GTP through interactions with PI3K subunit p85 or RAS-GTP. PIK3CA transcription in the central nervous system is regulated by microRNAs (miRNA). miRNA are endogenous single-stranded nonencoded small RNA, composed of 19~24 nucleotides, which can degrade the mRNA of target genes or inhibit their translation by completely or incomplete pairing with the 3′-nontranslational region of the target gene, thereby regulating the target gene expression at the posttranscriptional level. miR-124 is one of the most expressed miRNA in the mammalian nervous system and one of the most essential miRNA in neurological development [12], playing important roles in the development of the nervous system and synaptic plasticity. miR-124 knockout in rats leads to nervous system developmental maturation disorders [13]. miR-124 targets genes encoding proteins involved in a variety of biological processes such as neurological differentiation, learning, and memory. A study using the double fluorescein enzyme reporting system in rat neurons found that miR-124 specifically affects the 3′-UTR of PIK3CA [14], inhibiting its expression in the nervous system, further regulating the PI3K/Akt signaling pathway.

In a previous study, we found that La exposure decreases P-IKK expression and NF-*κ*B activity in the rat hippocampus [9]. NF-*κ*B is downstream of the PI3K/Akt signaling pathway, and it is unknown whether LaCl_3_ has regulatory effects on the PI3K/Akt signaling pathway. Here, we explore the effects of La exposure on learning and memory in newborn rats and in cultured HT22 cells to study the toxic effects of LaCl_3_ and analyze the molecular mechanism of LaCl_3_ neurotoxicity.

## 2. Materials and Methods

### 2.1. Preparation of Rats Exposed to LaCl_3_

Forty-eight Wistar rats (7–8 weeks old), 260 ± 10 g, half male and half female, were purchased from the Laboratory Animal Center of China Medical University, laboratory animal license No. SYXK-2003-0013. All of the animal experiments were approved by the Experimental Animal Welfare Ethics Committee of China Medical University (Approval No. CMU2018156). Rats were randomly divided into four groups: the control group, low-LaCl_3_ group (La-L group), medium-LaCl_3_ group (La-M group), and high-LaCl_3_ group (La-H group), with LaCl_3_ (Sinopharm, Peking, China) concentrations of 0.25%, 0.5%, and 1%, respectively, in the drinking water. Six pairs of rats were in each group, with male and female rats housed together with a 1 : 1 ratio. The day that a vaginal plug was observed was recorded as day 0 of pregnancy. Rats in each group were administrated La in their drinking water *ad libitum*. Rat offspring were exposed to La through the mother's blood circulation and breast milk before weaning; after weaning, offspring were administrated with La in drinking water at the same concentration of their mothers for 28 days. The weight and routine indicators of growth and development of pregnant rats and their offspring were recorded throughout the experiment. At the end of experiment, the rats were decapitated after anesthesia with pentobarbital sodium (40 mg/kg) to collect the brain tissues for further research.

### 2.2. Morris Water Maze Test

A learning and spatial memory behavior test was performed in a circular pool with a 150 cm diameter, 60 cm height, and 30 cm depth at 22 ± 2°C. Milk was added to make the water opaque. The barrel was divided into four quadrants, and a 15 cm diameter hidden platform was placed in the middle of the second quadrant of the pool 2 cm under the water surface. Rat offspring were put into the water at the midpoint of the four quadrants 4 times per day for 5 days to train. Time spent to find the platform was recorded. After 5 days' training, the rats were used for two tests: the positioning navigation experiment, which assessed the rat's escape incubation period and swimming distance (swimming path length), and the space exploration experiment, in which the platform was removed after 4 h at the end of the positioning navigation experiment, and rats were kept swimming in the pool for 60 s. Number of times entering the target quadrant, time spent in the target quadrant, and the search path were all recorded.

### 2.3. Detection of La Content in the Hippocampus

Rat hippocampal tissue, 10-80 mg weight, was stored in sealed Teflon tubes with 2 mL 65% nitric acid and 1 mL H_2_O_2_. Tubes were placed in a steel tank which was heated to 180°C for 4 h to digest the hippocampal tissue. Finally, 2% nitric acid was used to dilute the digested hippocampal tissue to 10 mL, and the La content in the hippocampus samples was detected by an inductively coupled plasma mass spectrometer (ICP-MS).

### 2.4. Hematoxylin and Eosin (H&E) Staining

Formalin-fixed tissue samples were progressively incubated in 70%, 80%, 90%, 95%, and 100% alcohol. Xylene was used to clear the sample. Samples were embedded into paraffin blocks, cut into 4 *μ*m sections, then dewaxed. Hematoxylin was stained for 5 min; then, slides were washed in PBS, dunked in 1% hydrochloric acid, stained with eosin for 30 seconds, then dehydrated with gradient alcohol. Neutral gum was used for sealing. Pathological changes in each group of tissue were observed under a light microscope.

### 2.5. TUNEL Assay

The brain tissue of rats was fixed in 4% polyformaldehyde. After embedding in paraffin, the tissue was cut into 3 *μ*m coronal slices. After dewaxing and hydrating by alcohol gradient, the slices were incubated with protease K at 25°C for 30 min to remove tissue proteins. The slices were put in 3% H_2_O_2_ at room temperature for 10 min in a dark room, washed with ddH_2_0 three times for 5 min, then washed in PBS for 5 min. 50 *μ*L of the TdT enzyme reaction buffer was added and incubated for 60 min at 37°C in a wet box. After washing with PBS, each sample received 50 *μ*L streptavidin-TRITC solution, then incubated in a wet box at 37°C for 30 min. Samples were then washed and stained with DAPI for 15 min, washed with PBS, and sealed. Samples were observed by a microscope. Positive cells were analyzed using ImageJ software.

### 2.6. Cell Culture and Drug Treatment

A mouse hippocampal cell line (HT22) was cultured in Dulbecco's modified Eagle's medium (DMEM) supplemented with 10% fetal bovine serum (FBS) and antibiotic/antimycotic (1%) in an incubator supplied with 5% CO_2_ at a temperature of 37°C. LaCl_3_ was dissolved in serum-free DMEM. Cells were randomly divided into six groups: control group, LaCl_3_ group (0.5 mM LaCl_3_), miR-124 inhibitor group, miR-124 mimic group, PIKI3CA high-expression group, and miR-124 mimic+PIKI3CA high-expression group. After reaching 70% confluency, cells were separately transfected with miR-124 inhibitor, miR-124 mimics, and PIK3CA overexpression vector or cotransfected with miR-124 mimics and PIK3CA overexpression vector (GenePharma, Shanghai, China). Transfections were performed using Lipofectamine 3000 reagent as the manufacturer's protocol (Thermo Fisher Scientific, MA, USA). After successful transfection, cells were treated with 0.5 mM LaCl_3_.

### 2.7. ELISA

The supernatant was collected, and the level of PSD 95 in the cell lysate was measured by ELISA according to the PSD95 kit (Shanghai Lengton Bioscience Co., Ltd., Shanghai, China).

### 2.8. Apoptosis Detection by Flow Cytometry Assay

Cells were trypsinized and washed with PBS; then, 1 × 10^6^ cells were resuspended in PBS. After adding 5 *μ*L of annexin V (556547, BD Pharmingen) and 5 *μ*L of propidium iodide (PI; 556547, BD Pharmingen), cells were incubated for 30 min at room temperature protected from light. The cells were then washed with PBS and resuspended with 300 *μ*L of PBS. The apoptosis ratio was calculated using FlowJo (BD Biosciences, USA).

### 2.9. Dual-Luciferase Reporter Gene Assay

Biological prediction websites including microRNA.Org (http://www.microrna.org/microrna/getGeneFormdo) and mirdb.Org (http://mirdb.org/) predicted PIK3CA as the target protein of miR-124. A synthetic PIK3CA 3′-UTR gene fragment was inserted into a pMIR-reporter using restriction enzyme sites SpeI and HindIII (Shenyang Wanlei Biotechnology Co., Ltd., Shenyang, China). The mutant complementary sequences were designed according to the sequence of wild-type PIK3CA, and the target fragment was inserted into the pMIR-reporter carrier by T4 DNA ligase after digestion with restriction endonucleases. WT and mutant (MUT) plasmids were transmitted to HEK-293T with miR-124 mimics or miR-nonsense control (miR-NC), respectively. After 48 h cultivation, cells were collected and separated, and signal was detected with the luciferase detection kit (Promega, USA).

### 2.10. Real-Time PCR

Total RNAs from brain tissue and neuron cells were extracted using TRIzol. 100 mg brain tissue or 1 × 10^8^ cells were treated with 1 mL TRIzol. cDNA was synthesized according to the kit instructions. 2 *μ*L of cDNA was used as a template for PCR, in accordance with qRT-PCR instructions, using *gapdh* as a reference gene. DNA primer sequences are shown in Table 1. Relative expressions were calculated by the 2 ^−*ΔΔ*CT^ method. The reaction conditions were as follows: 95°C for 5 min, 94°C for 30 s, 55°C for 30 s, 72°C for 60 s, 30 cycles, and 72°C for 1 min.

### 2.11. Western Blotting

Proteins were extracted from brain tissue and neuronal cells. Brain tissue was cut into pieces, and cultivated cells were scraped from their plate. The tissue or cells were homogenized in RIPA lysate containing protease inhibitor at 4°C, then centrifuged at 12000 rpm. After 20 min, the supernatant was collected and the protein concentration determined using the BCA Protein Assay Kit (Thermo Fisher Scientific, USA). A 10% SDS-PAGE gel was prepared; then, 30 *μ*g of each protein sample was loaded on the gel, separated by electrophoresis, and transferred to PVDF membrane. The membrane was blocked by 5% skim milk solution for 1.5 h and washed by TBST 3 times for 5 min each. The membrane was then separately incubated with primary antibodies against PIK3CA, PI3K, p-Akt, p-NF-*κ*B p65, Akt, NF-*κ*B p65, or GAPDH (1 : 1000, Abcam, Cambridge, MA, USA) at 4°C overnight. After being washed by TBST, the membrane was incubated with HRP-labeled secondary antibodies (1 : 1000, Santa Cruz, USA) for 1 h at room temperature. After washed by TBST, the membrane was illuminated by the ECL luminescence kit, imaged with gel imaging system. ImageJ software was used to calculate the gray value. The optical density ratio of the target protein band and the internal reference protein band was used for statistical analysis.

### 2.12. Statistical Analysis

All experiments were performed in at least three independent repetitive trials. SPSS 18.0 software was used for statistical analysis. The results were represented by mean ± SD. One-way ANOVA was used for the significance analysis. When the variance was equal, the least significant difference (LSD) method was used for the pairwise comparison. When the variance was not uniform, the Tamhane T2 method was used for the pairwise comparison. *P* < 0.05 was considered statistically significant.

## 3. Results

### 3.1. La Exposure Could Impair Spatial Memory and the Avoidance Response and Decrease Learning and Memory Capabilities

**Figure 1:**
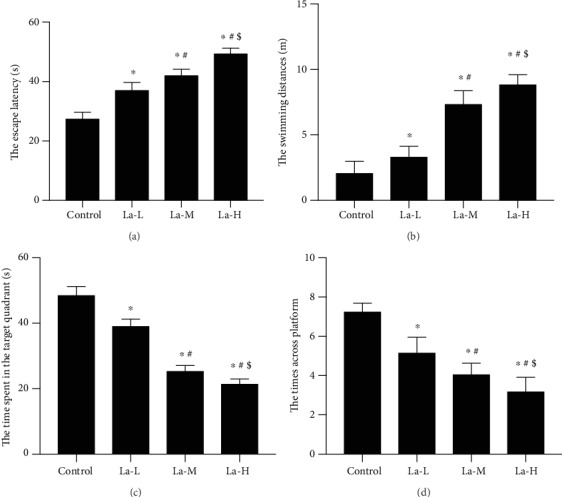
La exposure could impair spatial memory and the avoidance response and decrease learning and memory capabilities. (a) The escape incubation period of rats in each group; (b) the swimming distance of rats in each group; (c) the time rats in each group stayed in the target quadrant; (d) number of times rats in each group entered the target quadrant. ^∗^*P* < 0.05 compared with the control group.

To investigate the effect of La on learning and memory capability of rats' offspring, rat breeding pairs and their offspring were treated with low, medium, or high levels of LaCl_3_. We then used Morris water maze to detect behavioral changes in each group of rats' offspring. In the location navigation experiment, the escape incubation period of rats in the La-L, La-M, and La-H groups was significantly prolonged compared with that in the control group (Figure 1(a), *P* < 0.05) and the swimming distance of rats in each dose group was also significantly extended (Figure 1(b), *P* < 0.05). In spatial exploration experiments, time in the target quadrant was significantly shorter for the La-M and La-H groups than that in the control group (Figure 1(c), *P* < 0.05). The La-L, La-M, and La-H groups all entered the target quadrant significantly less than the control group as well (Figure 1(d), *P* < 0.05). Moreover, with an increase in LaCl_3_ dose, the offspring rats looking for the underwater platform were disorganized, the escape latency increased (Figure 1(a), *P* < 0.05), and the swimming distance got longer (Figure 1(b), *P* < 0.05). Additionally, increased doses of LaCl_3_ decreased the time spent in the target quadrant of offspring rats (Figure 1(c), *P* < 0.05) and reduced the times of passing through the underwater platform (Figure 1(d), *P* < 0.05).

### 3.2. La Exposure Led to Significantly Increased La Content in the Hippocampus of Rats' Offspring

**Figure 2:**
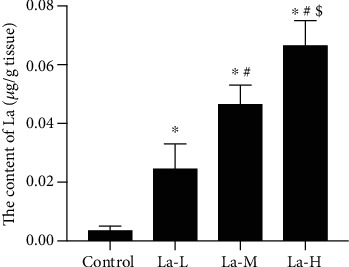
La exposure led to a significant increase in La content in the hippocampus of the offspring of treated rats. La content was detected in the hippocampus of offspring rats. ^∗^*P* < 0.05 compared with the control group.

To determine whether the learning defects seen above were caused by La physically affecting brain development, we measured the La content in the hippocampus of the La-treated rats' offspring. The content of La in the hippocampus of rats' offspring in the La-L group was significantly higher than that in the control group; the La content in the La-M group was significantly higher than that in the La-L group; moreover, the La content in the La-H group was significantly higher than that in the La-M group. Thus, La accumulation in the hippocampus of the rats' offspring positively correlated with La exposure level (Figure 2, *P* < 0.05).

### 3.3. La Exposure Impaired the Synaptic Structure of Hippocampal Neurons in the Offspring of Treated Rats and Increased Neuronal Apoptosis

**Figure 3:**
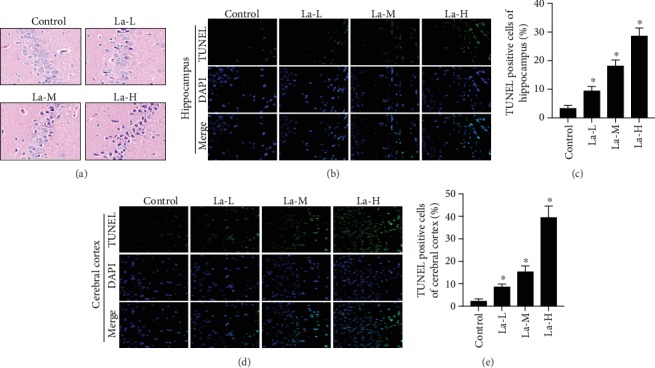
La exposure impaired the synaptic structure of hippocampal neurons and increased neuronal apoptosis in the offspring of treated rats. (a) H&E staining (scale bar = 50 *μ*m); (b) TUNEL assay of the hippocampus (scale bar = 50 *μ*m); (c) quantification of the TUNEL assay in the hippocampus; (d) TUNEL assay of the cortex (scale bar = 50 *μ*m); (e) quantification of the TUNEL assay in the cortex; ^∗^*P* < 0.05 compared with the control group.

As shown in Figure 3(a), H&E staining revealed that hippocampal neurons in the control group were arranged in a regular and tight manner with clear cell boundaries and intact cell bands. Only a small number of inflammatory cells were present. In offspring of rats treated with low, medium, and high La concentrations, the hippocampus exhibited severe damage and nerve cells were disordered. Neuron and cone cell death was observed, and cell numbers were significantly decreased in the hippocampus. We used TUNEL staining to determine the apoptosis rates of nerve cells in the cortex of each group of offspring. Compared with the control group, the number of apoptotic nerve cells in the hippocampus and cortex gradually increased in offspring of rats treated with low, medium, and high concentrations of La.

### 3.4. La Increased miR-124 Levels and Inhibited Expression of PIK3CA, PI3K, p-Akt, and p-NF-*κ*B p65 Proteins

**Figure 4:**
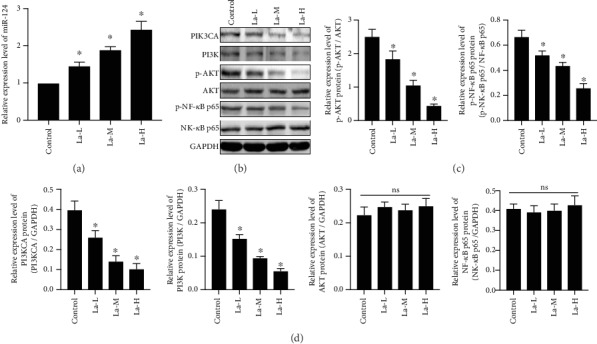
La could increase miR-124 levels and inhibit the expression of PIK3CA, PI3K, p-Akt, and p-NF-*κ*B p65 proteins. (a) Detection of the expression of miR-124 by real-time PCR; (b) PIK3CA, PI3K, p-Akt, Akt, p-NF-*κ*B p65, and NF-*κ*B p65 protein expression was detected by western blot; (c, d) quantification of the western blot; ^∗^*P* < 0.05 compared with the control group; *ns*: not significant, indicates *P* > 0.05.

miR-124 was reported to regulate synaptic plasticity and neuronal apoptosis, influencing learning and memory [13, 14]. Therefore, we used real-time PCR to detect miR-124 expression in our rat offspring model. Compared with the control group, miR-124 expression in the brain tissue gradually increased across the offspring of rats treated with low, medium, and high La concentrations (*P* < 0.05, Figure 4(a)). This suggests that La increases miR-124 expression in the brain tissue of rat offspring, presenting a possible mechanism for neuron and synapse injury. To identify whether La-induced toxic damage in hippocampal neurons is related to PI3K/Akt signaling pathway regulation by the miR-124 target PIK3CA, we detected the expression of PI3K/Akt signaling pathway-related proteins PIK3CA, PI3K, p-Akt/Akt, and p-NF-*κ*B p65/NF-*κ*B p65. Compared to the control group, the expressions of PIK3CA, PI3K, p-Akt, and p-NF-*κ*B p65 were all significantly reduced in the brain tissue of the offspring rats (*P* < 0.05, Figures 4(b)–4(d)), indicating that La leads to hippocampal neurotoxicity through regulating miR-124 expression to inhibit the PI3K/Akt pathway.

### 3.5. La Decreased Expression of PSD95 in Neurons through Upregulating miR-124

**Figure 5:**
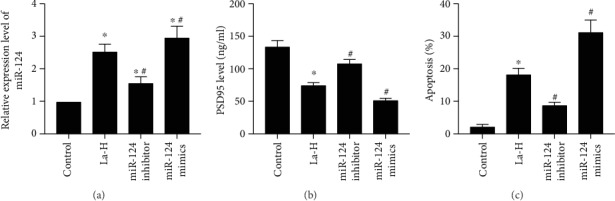
La decreased expression of PSD95 in neurons through upregulating miR-124, and La exposure could promote neuronal apoptosis, which could be rescued by a miR-124 inhibitor. (a) Expression levels of miRNA-124 detected by real-time PCR; (b) PSD95 in hippocampal neurons detected by ELISA; (c) cell apoptosis detected by flow cytometry; ^∗^*P* < 0.05 compared with the control group; ^#^*P* < 0.05 compared with the La-H group.

To explore the relationship among La exposure, neuronal injury, and miR-124, we transected miR-124 inhibitors or mimics into HT22 cells cultured in vitro. To observe the effect of La exposure on synaptic function of hippocampal neurons in rats, the synaptic protein marker postsynaptic density protein 95 (PSD95) was examined in hippocampal neurons by ELISA (Figures 5(a) and 5(b)). The results showed that PSD95 expression decreased significantly in La-exposed cells compared to the control group. PSD95 expression increased significantly after transfection with miR-124 inhibitor, and the expression of PSD95 decreased significantly after transfection of miR-124 mimics, indicating that La exposure is related to synaptic injury and miR-124 regulation.

### 3.6. La Exposure Increased miR-124 Levels to Promote Neuronal Apoptosis, Which Could Be Rescued with an miR-124 Inhibitor

To observe the effect of La on apoptosis in hippocampal neurons, we used flow cytometry to detect neuronal apoptosis. We found increased neuronal apoptosis in the La-exposed group (*P* < 0.05) compared to the control group, and neuronal cell apoptosis decreased significantly after transfection with a miR-124 inhibitor (*P* < 0.05, Figure 5(c)). Neuronal cell apoptosis increased significantly following transfection with miR-124 mimics, indicating that La exposure may lead to neuronal apoptosis through a mechanism related to regulation of miR-124 expression.

### 3.7. PIK3CA Has Been Shown to Be Targeted by miR-124

**Figure 6:**
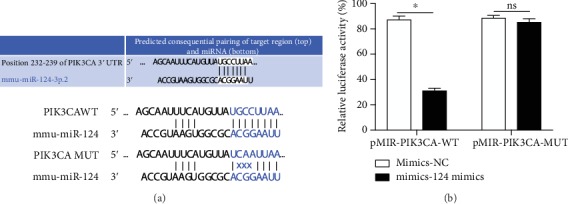
PIK3CA is one of the targeting proteins of miR-124. (a) Target prediction of miRNA-124; (b) dual-luciferase reporter assay; ^∗^*P* < 0.05; *ns*: not significant, indicates *P* > 0.05.

To figure out the target protein of miR-124, firstly, we predicted the targeting using http://microrna.org/ and http://mirdb.org/. We found that PIK3CA is one of the predicted target proteins of miR-124 and identified a 3′-UTR specific binding area between the sequence of PIK3CA and miR-124 (Figure 6(a)). Moreover, we successfully transfected a pMIR-PIK3CA-WT vector or pMIR-PIK3CA-MUT in HEK-292T cells; after, the miR-124 or mimic NC was transfected into two types of cells. The dual-luciferase reporter assay demonstrated that compared to the mimic NC group, miR-124 significantly inhibited luciferase activity (*P* < 0.05) in pMIR-PIK3CA-wt-3′-UTR transfected cells, but it had no effect on the fluorescein enzyme activity in pMIR-PIK3CA-mut-3′-UTR cells (Figure 6(b)). This result indicated that miR-124 can inhibit PIK3CA expression by directly binding the target sequence on the 3′-UTR of the PIK3CA gene.

### 3.8. La Exposure Causes Neurotoxicity in Rats by Upregulating miR-124 Expression Which Targets PIK3CA to Regulate the PI3K/Akt Signaling Pathway

**Figure 7:**
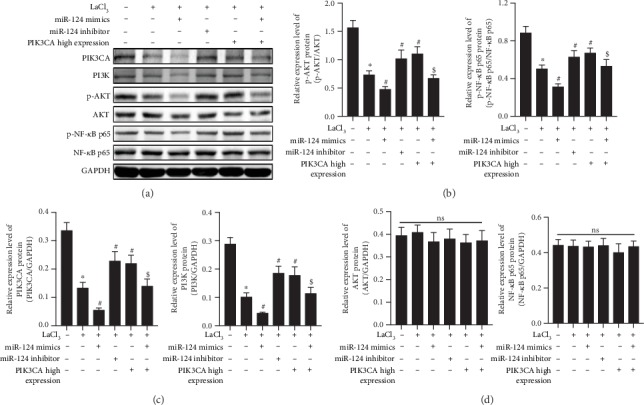
La chloride causes neurotoxicity in rats by upregulating miR-124 expression, which targets PIK3CA to regulate the PI3K/Akt signaling pathway. PIK3CA, PI3K, p-Akt, AKT, p-NF-*κ*B p65, and NF-*κ*B p65 protein expression was detected by western blot; (c, d) Quantifications of the western blot; ^∗^*P* < 0.05 compared with the control group; ^#^*P* < 0.05 compared with the La-H group; ^$^*P* < 0.05 compared with the PIC3K high-expression group; *ns*: not significant, indicates *P* > 0.05.

To investigate the relationship between La exposure and the PI3K/Akt signaling pathway, we used western blotting to detect expression of PIK3CA, PI3K, p-Akt, and p-NF-*κ*B p65 proteins in cultured HT22 cells with or without La exposure. As shown in Figures 7(a)–7(c), La caused a significant downregulation of PIK3CA, PI3K, p-Akt, and p-NF-*κ*B p65 protein expression (*P* < 0.05), and the effect could be blocked with miR-124 inhibitors (*P* < 0.05). Moreover, miR-124 mimics could enhance the inhibitory effect of La exposure on PIK3CA, PI3K, p-Akt, and p-NF-*κ*B p65 protein levels (*P* < 0.05). Compared to the LaCl_3_ group, the expression of PI3K, p-Akt, and p-NF-*κ*B p65 in HT22 cells was upregulated in the PIK3CA overexpression group; interestingly, the increased expression of PI3K, p-Akt, and p-NF-*κ*B p65 could be blocked in the miR-124 mimics+PIK3CA overexpression group. These results suggested that La exposure may cause neurotoxicity through activating miR-124, which inhibits PIK3CA to regulate the PI3K/Akt signaling pathway.

## 4. Discussion

REEs are nonessential elements for the human body which can cause multisystem, multiorgan damage at certain doses. Studies have shown that REEs have neurological toxicity and affect learning and memory capability [1, 2]. The hippocampus is located in the basal part of the inner temporal lobe of the brain and is an important part of the limbic system. The hippocampus is an important tissue in information processing and is closely related to learning and memory [15]. The results of our study showed significant damaged learning and memory abilities in rats following exposure to LaCl_3_. Rat pups exposed to LaCl_3_ through one month after weaning showed La accumulation in the hippocampus which positively correlated with the LaCl_3_ exposure level. This accumulation in the hippocampus may be the basis for the effects of La on the structure and function of the central nervous system.

PSD95 is an important protein on the posterior synaptic membrane skeleton that is necessary for maintaining the activity and stability of synapses and regulating their plasticity [16–18]. In this study, we detected the expression of PSD95 in HT22 cells in vitro. We found that PSD95 expression in neuronal cells decreased with LaCl_3_ injury, compared with the control group, indicating that La can reduce synaptic plasticity.

Cell apoptosis is a programmed death of cells under the control of their own genes, and it is an essential physiological process in the development of the mammalian nervous system. However, in the presence of harmful physical and chemical factors, nerve cells usually experience high rates of “pathological” apoptosis, decreasing neuron number and significantly reducing neuronal function and efficiency [19]. As neurons have poor regeneration capacity, the effects of this injury on the development and function of the nervous system may be long term and irreversible. Studies have shown that LaCl_3_ can increase the concentration of calcium ions in rat hippocampal neuronal cells, leading to mitochondrial dysfunction and excessive reactive oxygen species (ROS), much like the ability of La to reduce Nrf2/ARE signaling pathways and expression of antioxidant proteins causing astrocyte damage [1, 20]. In this study, we analyzed neuronal apoptosis in vivo and in vitro after La exposure and found that apoptosis significantly increased under LaCl_3_ exposure, indicating that La can cause significant neurotoxicity in vivo and in vitro.

To further explore the molecular mechanism of LaCl_3_-induced neurotoxicity, we analyzed miR-124 expression. MicroRNAs are evolutionary conserved noncoding RNAs. miR-124 is specifically expressed and enriched in brain tissue, regulating the expression of at least one-third of the human genome and playing key roles in a variety of biological processes including cell differentiation, apoptosis, development, and metabolism [20–22]. As miR-124 is not expressed by astrocytes, it is considered to be neuron-specific [23]. In this study, we first analyzed miR-124 expression in the brain tissue of La-exposed rats and found that increasing concentrations of LaCl_3_ lead to dose-dependent elevation of miR-125 expression in the hippocampus of rats. In cultured neurons, adding LaCl_3_ significantly increased miRNA expression. More interestingly, when we transfected miR-124 inhibitors or mimics into HT22 cells in vitro, the miR-124 inhibitor rescued the effect of increasing miR-124 expression by LaCl_3_. On the contrary, miRNA-124 mimics acted synergistically with LaCl_3_, leading to significantly increased miR-124 expression. We then analyzed the regulation of miR-124 expression on PSD95 expression and apoptosis in HT22 cells and found that miR-124 inhibitors could reverse the LaCl_3_-induced reduction of PSD95 expression. However, miRNA-124 mimics were shown to reduce PSD95 expression in HT22 cells damaged by LaCl_3_, with similar effects on apoptosis rate. These results fully indicate that LaCl_3_ neurotoxicity is caused by upregulation of miR-124 expression.

The PI3K/Akt pathway is an important antiapoptosis signaling pathway regulated by a variety of inflammatory factors [24]. In the central nervous system, miR-124 regulates PIK3CA transcription, which in turn activates PI3K. After PI3K activation, the second messenger 3,4,5-three phosphoric acid phosphatidylcholine (PIP3) is produced on the membrane. PIP3 can bind with signal protein Akt containing the PH domain, phosphoinositide-dependent kinase-1 (PDK1), and phosphoinositide-dependent kinase-2 (PDK2). PIP3 then phosphorylates Ser308 and Thr473 to activate Akt [25]. Phosphorylated Akt activates transcription factor NF-*κ*B, which promotes cell proliferation and inhibits cell apoptosis upon entering the nucleus. Studies have shown that hippocampal long-term potentiation (LTP) was significantly inhibited in an animal model of Alzheimer's disease and that this inhibition is closely related to Akt1 [26]. Recent studies have reported that nerve damage and LTP inhibition caused by iodine deficiency in rats are closely related to the PI3K/Akt signaling pathway [27]. In a previous study, we found that La exposure led to decreased P-IKK expression in the hippocampus and reduced NF-*κ*B activity [9]. As NF-*κ*B is an important downstream protein of the PI3K/Akt signal pathway, this reduction suggested that the PI3K/Akt signal pathway was abnormal following La exposure. In this study, PIK3CA was shown to be the direct target gene of miR-124. In vivo and in vitro experiments showed that LaCl_3_ could decrease the expression of PIK3CA, PI3K, p-Akt, and p-NF-*κ*B p65 proteins compared with the control groups. We also observed that the regulatory function of LaCl_3_ on PI3K/Akt signaling pathway-related proteins can be blocked by a miR-124 inhibitor or enhanced by miR-124 mimics. Compared with the LaCl_3_-exposed group, not only were PIK3CA, PI3K, p-Akt, and p-NF-*κ*B p65 proteins increased in neuronal cells with highly expressed PIK3CA, but the expression of SYN and PSD-95 were also increased significantly and LaCl_3_-induced apoptosis was significantly reduced, all of which could be reversed by miR-124 mimics.

## 5. Conclusions

In this study, we reported that lanthanum chloride caused the injury and neurotoxicity on the hippocampus of offspring rats and firstly identified that the mechanism of injury depended on upregulating miR-124 expression and targeting PIK3CA through the PI3K/Akt signaling pathway. Therefore, overexpression of miR-124 and downregulation of PIK3CA induced by La may be an important clue of La-induced neuron impairment.

## Figures and Tables

**Table 1:** The primer sequences of miR-124 and U6.

Gene name	The primer sequences (5′⟶3′)
miR-124 upstream primer	GCTAAGGCACGCGGTG
miR-124 downstream primer	GTGCAGGGTCCGAGGT
U6 upstream primer	CTCGCTTCGGCAGCACA
U6 downstream primer	AACGCTTCACGAATTTGCGT

## Data Availability

The datasets used and/or analyzed during the current study are available from the corresponding author upon reasonable request.
